# Enhanced magnetic modulation of light polarization exploiting hybridization with multipolar dark plasmons in magnetoplasmonic nanocavities

**DOI:** 10.1038/s41377-020-0285-0

**Published:** 2020-03-30

**Authors:** Alberto López-Ortega, Mario Zapata-Herrera, Nicolò Maccaferri, Matteo Pancaldi, Mikel Garcia, Andrey Chuvilin, Paolo Vavassori

**Affiliations:** 1 CIC nanoGUNE - BRTA, Donostia–San Sebastian, Donostia, 20018 Spain; 2 Department of Physics and Materials Science, University of Luxembourg, L-1511 Luxembourg, Luxembourg; 3 Department of Physics, Stockholm University, 106 91 Stockholm, Sweden; 4 IKERBASQUE, Basque Foundation for Science, Bilbao, 48013 Spain

**Keywords:** Nanocavities, Magneto-optics

## Abstract

Enhancing magneto-optical effects is crucial for reducing the size of key photonic devices based on the non-reciprocal propagation of light and to enable active nanophotonics. Here, we disclose a currently unexplored approach that exploits hybridization with multipolar dark modes in specially designed magnetoplasmonic nanocavities to achieve a large enhancement of the magneto-optically induced modulation of light polarization. The broken geometrical symmetry of the design enables coupling with free-space light and hybridization of the multipolar dark modes of a plasmonic ring nanoresonator with the dipolar localized plasmon resonance of the ferromagnetic disk placed inside the ring. This hybridization results in a low-radiant multipolar Fano resonance that drives a strongly enhanced magneto-optically induced localized plasmon. The large amplification of the magneto-optical response of the nanocavity is the result of the large magneto-optically induced change in light polarization produced by the strongly enhanced radiant magneto-optical dipole, which is achieved by avoiding the simultaneous enhancement of re-emitted light with incident polarization by the multipolar Fano resonance. The partial compensation of the magneto-optically induced polarization change caused by the large re-emission of light with the original polarization is a critical limitation of the magnetoplasmonic designs explored thus far and that is overcome by the approach proposed here.

## Magneto-optics: nanocavity enhancement

Magnetic control of light can be greatly enhanced by custom-designed magnetoplasmonic nanocavities, report European scientists. Alberto López-Ortega and coworkers from Spain, Luxembourg and Sweden fabricated arrays of plasmonic gold-ring nanoresonators that feature a ferromagnetic nanodisk located asymmetrically within them. The coupling and hybridization of multi-polar dark modes and the dipolar plasmon resonance within the nanocavity results in a large amplification of the cavity’s magneto-optical response. As a result, the polarization of re-emitted light can be controlled via modulation of a weak external magnetic field. The approach may ultimately enable the construction of efficient nanophotonic devices for performing polarization-based tasks such as light isolation, rotation, modulation and sensing. In the future, the implementation of the concept using different ring materials and an optimized geometry could bring further and substantial improvements in the strength and tunability of the magneto-optical effects.

## Introduction

Nanophotonics uses light polarization as an information carrier in optical communications, sensing, and imaging^1^. Likewise, the state of polarization plays a key role in the photonic transfer of quantum information^2^. In this framework, optical nanodevices enabling the dynamic manipulation of light polarization at the nanoscale are key components for future nanophotonic applications. The electromagnetic interaction with plasmonic meta-surfaces and crystals has emerged as a prominent route to developing more efficient devices for the active control of light at sub-wavelength scales^3–5^. One relevant example is the class of magnetoplasmonic surfaces and crystals, composed of arrangements of nanoantennas either entirely^6–12^ or partially^13–20^ made of magnetic materials. For other magnetoplasmonic designs, researchers explored the integration of plasmonic nanostructures with dielectric^21,22^ or ferrimagnetic garnet^15,23^ films, as well as the integration of magnetic films in surface plasmon polariton waveguides and cavities^24,25^. Magnetic materials exhibit the so-called magneto-optical (MO) activity, arising from spin-orbit coupling of the electrons, which results in a weak magnetic-field-induced intensity and polarization modulation of reflected and transmitted light (on the order of 0.1°). The unique optical properties of magnetoplasmonic nanoantennas arise from combining strong local enhancements of electromagnetic fields (up to ten times) via localized plasmon excitations with their inherent MO activity. Additionally, light propagation in the presence of magneto-optical materials becomes non-reciprocal due to the time-reversal symmetry breaking caused by magnetization, which is an axial time-odd vector field. Therefore, in the past decade, materials based on magnetoplasmonic nanoantennas have been intensively investigated for their enhanced MO and non-reciprocal light propagation properties, aiming for 2D flat-optics nanodevices, such as rotators, modulators, and isolators^8,10,13–19,26–29^, as well as for their accuracy in the measurement of distances at the nanoscale^30^ and very small refractive index changes in label-free biosensing applications^7,11,31–35^.

To date, most studies of magnetoplasmonic nanostructures have exploited MO enhancement produced by bright (radiative) plasmon modes, such as localized dipolar plasmonic resonances (LPRs)^6–19^. For a circular disk-like magnetoplasmonic nanoantenna, the mechanism leading to the amplification of its MO response is well understood^36^. A linearly polarized incident radiation of proper wavelength excites a dipolar LPR in the nanoantenna that re-irradiates light with the same incident polarization. The application of an external magnetic field ***H*** induces a net magnetization ***M*** in the nanoantenna that turns on the MO activity in the material. In this situation, the LPR drives a second, MO-induced LPR in a direction orthogonal to both ***M*** and the LPR^36^. The radiation re-emitted by this second MO-induced LPR (MO-LPR) is polarized in a direction perpendicular to that of the incident radiation and determines the magnetic-induced polarization change. More specifically, the ratio of the amplitudes and the phase lag between these two orthogonal resonant bright electric dipoles determines the magnetic-field-induced polarization change of interacting light^36^. The amplitude of the electric dipole associated with the LPR is approximately Q times larger than that induced in the continuous film counterpart, where Q is the quality factor of the resonance. Since the MO-LPR is driven by the LPR, the amplitude of the associated electric dipole is approximately Q^2^ times larger than that in the continuous film. However, as we mentioned above, the resulting MO effect, i.e., the resulting magnetic-induced polarization change, is proportional to the ratio between the MO-LPR and LPR amplitudes^36^, limiting the maximum achievable enhancement of the MO activity to only a factor Q. This Q-fold enhancement of the MO response, therefore, represents an unsurmountable upper limit achievable with metallic magnetoplasmonic nanoantennas.

For typical ferromagnetic metallic constituents, both the LPR and MO-LPR have a low Q-factor relative to that of noble metals. A Q-factor on the order of 10 is typical of Au and Ag nanostructures in the visible–near-infrared spectral range^37^, while the Q-factor of ferromagnetic nanoantennas in the same spectral range is on the order of 3–4, depending on the material, shape, and size of the nanoantenna^6,7^. Higher Q-factor values, approaching that of noble metals, can be obtained using multilayered ferromagnetic/noble metal nanoantennas^38^. Therefore, the maximum achievable enhancement of the MO activity can be up to only ~ 1-order of magnitude using the magnetoplasmonic nanoantenna designs explored thus far^6–19^. This Q-limited enhancement of MO activity triggered the exploration of different geometries such as, for example, heterogeneous noble and ferromagnetic vertical dimers^39–41^ and split-ring resonators combining plasmonic and magnetic materials, with the latter as an integrating part of the ring^29,42^. Although these systems displayed resonances with an improved Q-factor, the physics governing their electrodynamics connected to the MO activity still relies on the excitation of bright plasmons as in conventional magnetoplasmonic structures.

Since the Q-limited enhancement of the MO activity arises from the re-irradiations of light with the incident original polarizations, we directed our attention to systems that can support dark modes and their hybridization with bright modes, which should lead to the generation of low-radiant hybrid modes. Archetypical structures investigated in the literature are symmetric nanorings and concentric ring/disk nanocavities^43–47^. These nanocavities can sustain multipolar dark modes, but the symmetry of their charge distribution forbids direct coupling to free-space photons at normal incidence. Excitation of the dark modes is enabled when the rotational symmetry of the unit is broken, e.g., by displacing the disk position away from the ring centre. These non-concentric ring/disk nanocavities lead to the appearance of new modes corresponding to Fano interferences generated by the hybridization of the dipolar mode of the disk and the multipolar dark modes of the ring^43,44^.

However, these previously studied nanocavities did not possess any MO properties since they were synthesized from non-magnetic plasmonic materials, typically gold^43,44^. Therefore, the implementation of magnetoplasmonics in nanocavities remains an unexplored terrain. In this study, we propose a design and achieve the fabrication of bi-component magnetoplasmonic nanocavities composed of a gold ring plasmonic resonator and an MO-active ferromagnetic nanodisk asymmetrically placed inside the ring. We explore here the unique potential of this construct for enhancing and controlling the polarization of re-emitted light via modulation of an external magnetic field.

We observe that incident linearly polarized light excites a Fano resonance resulting from the hybridization of the LPR in the magnetic disk with a multipolar dark mode in the plasmonic ring. This hybridized multipolar mode results in low radiation and thus does not significantly enhance the re-emission of light with primary polarization. In turn, when the magnetic nanodisk is magnetically activated under the application of a magnetic field ***H*** parallel to the wavevector of the incident electromagnetic wave, the hybrid low-radiant mode drives an intense and bright, i.e., highly radiant, MO-LPR in the ferromagnetic nanodisk orthogonal to the incident polarization. Notably, the MO-LPR driven by the hybridized multipolar mode is much more intense (~1 order of magnitude) than the MO-LPR achievable in bare magnetoplasmonic nanoantennas. The suppression of the detrimental effect of the enhanced re-emission of light with the original polarization by replacing the LPR with the hybrid multipolar mode while simultaneously enhancing the MO-LPR, which is not hybridized and retains its dipolar bright character, produces an amplification of the MO response that goes beyond the Q-factor limit.

## Results

**Fig. 1:**
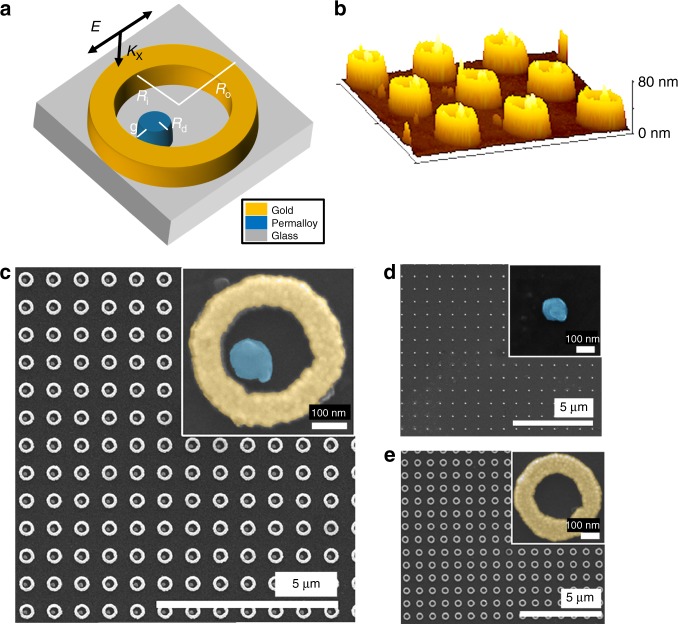
Magnetoplasmonic NCRD ferromagnetic-nanoantenna/gold nanocavity and parent Py-DI and Au-RI nanostructures. **a** Schematic of the NCRD hybrid structure with its four geometric characteristic parameters. **b** Atomic force and **c** SEM images of the individual NCRD nanocavity and array. SEM images of the parent single **d** Py-DI and **e** Au-RI constituents and arrays

A schematic of the magnetoplasmonic non-concentric ring disk (NCRD) nanocavity together with atomic force and scanning electron microscopy images of an array of these nanocavities are depicted in Fig. 1a–c. The Au ring is characterized by inner and outer ring radii of *R*_i_ = 130 nm and *R*_o_ = 215 nm, respectively; the radius of the ferromagnetic disk is *R*_d_ = 50 nm, and the gap between the disk and the ring is g ≈ 10 nm. The material utilized for the disk is Permalloy (Py), an Fe_20_Ni_80_ alloy. Py was chosen because, similar to Ni, its plasmonic, magnetic, and MO properties are well known. For comparison, arrays of isolated Py disk (Py-DIs) magnetoplasmonic nanoantennas and isolated Au rings (Au-RIs) have also been fabricated for reference and are depicted in Fig. 1d, e. We also fabricated a control sample with an array of concentric ring/disk (CRD) nanocavities using the same *R*_i_, *R*_o_, and *R*_d_ (Fig. S6). All the structures are 40-nm-thick and are arranged in a square array with a 780-nm pitch. The synthesis was carried out by electron-beam lithography, followed by electron-beam and thermal evaporation of the Py and Au materials, respectively, onto Pyrex substrates. Specifically, the synthesis of hybrid Py/Au nanocavities has been achieved by a finely controlled two-step electron-beam lithography process to sequentially grow the Au rings in the desired position around the pre-existing Py disks.

**Fig. 2:**
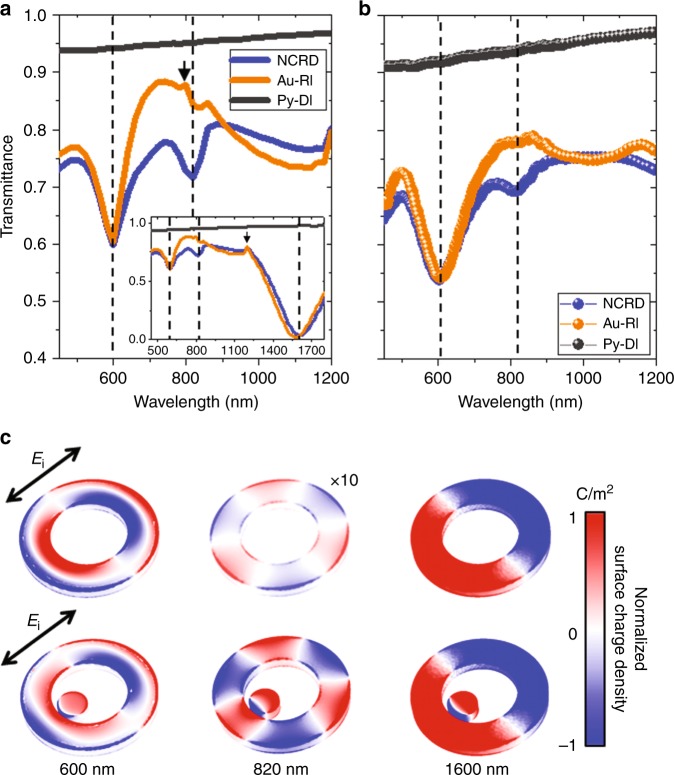
Au-RI and NCRD optical properties and modes. **a** Simulated and **b** experimental transmittance spectra for the NCRD, Py-DI and Au-RI structures. Dashed lines mark the major features in the spectra at 600, 820 and 1600 nm. The small black arrow in (**a**) highlights a minor feature due to the weak far-field diffractive coupling in a simulated periodic array of defect-less structures. **c** Surface charge density maps (see Materials and methods) for the Au-RI and NCRD structures at 600, 820 and 1600 nm, normalized to the map at 820 nm for the NCRD for direct comparison. The simulations in (**c**) were carried out using linearly polarized electromagnetic radiation, as indicated by the black arrow (*E*_*i*_ *=* 1 Vm^−1^). The surface charge density for the Au-RI at 820 nm was multiplied by a factor of 10 for visualization purposes

Simulated and experimental transmittance spectra for the studied structures are depicted in Fig. 2a, b. Both the experimental and simulated spectra of the NCRD array display two strongly marked dips located at 600 and 1650 nm and a weaker dip at 820 nm. A comparison with the spectra (simulated and experimental) of the array of bare Au-RIs and a close inspection of the spectral dependence of the calculated surface charge distribution maps in Fig. 2c reveal that the two most prominent dips in the NCRD spectrum correspond to the excitation of the so-called antibonding and bonding dipolar plasmonic resonances in the Au-ring portion of the nanocavity at 600 nm and 1650, respectively (see also Fig. S1)^43^. As featured in Fig. 2c and S1, the lower-wavelength antibonding resonance corresponds to a dipolar mode through the inner and the outer lateral surfaces of the ring; the bonding mode at 1600 nm corresponds to a dipolar resonance involving the entire ring structure. Both resonances are bright modes that can be excited by direct coupling with free-space light even in symmetric structures and thus appear in both the NCRD and Au-RI spectra. The presence of the Py disk nanoantenna in the NCRD only marginally perturbs these modes, which occur at roughly the same wavelength and with almost identical features in both the NCRD and Au-RI spectra. It is worth noting that far-field diffractive coupling due to the periodic array design of the samples, which could lead to the occurrence of the so-called surface lattice resonances (SLRs), produces extremely weak features in the experimental optical spectra. This is a common and known effect due to the non-homogeneous embedding medium, which strongly suppresses the strength of SLRs. The simulated spectra shown in Fig. 2a indeed show these features at 800 nm and at 1200 nm due to diffractive coupling through air and glass substrates, respectively (small black arrows in Fig. 2a). The array with standalone Py disks produces a very broad plasmonic dipolar resonance that peaks at ~550 nm (black solid lines in Fig. 2a, b)^6^. Indeed, plasmonic dipolar resonances in ferromagnets, which are lossy metals, are very broad and can typically extend over 350 nm (FWHM), as observed experimentally^6,7,36^.

A comparison of surface charge distribution maps of the isolated Au-RI and the NCRD nanocavity at 820 nm (Fig. 2c) clearly reveals that the dip at this wavelength arises from the strong and localized near-field coupling between the broad dipolar resonance of the Py disk with a high-order multipolar dark mode possessing a ***S***_*6*_ reflection-rotational (6-fold improper rotation) symmetry in the Au-ring portion of the nanocavity. In the literature, this mode is referred to as either the hexapolar or the octupolar mode of a ring^43,44^. Hereafter, we label this mode as ***S***_***6***_ in reference to the point-group notation. An inspection of Fig. 2c shows that the surface charge distribution of the Au-RI displays a very weak ***S***_***6***_ at 820 nm (note that the intensity of the surface charge density map had to be multiplied by a factor of 10 to become visible in Fig. 2c), while for the NCRD, the excitation of an intense ***S***_***6***_ mode is clearly visible, although it is slightly distorted due to hybridization with the dipole in the Py disk.

To experimentally confirm the complete plasmonic spectrum of the Au nanoring resonator, we performed an electron energy loss spectroscopy study using a scanning transmission electron microscope (STEM-EELS). For this purpose, we fabricated Au nanorings with the same dimensions as those of our NCRD nanocavities on a 20-nm-thick SiN_x_ membrane. The localized excitation realized in STEM-EELS^48^ can efficiently excite all eigenmodes supported by a plasmonic structure, including non-radiative modes; however, due to the rotational symmetry of the rings, the spatial distribution of the modes cannot be visualized. Complementarily, we conducted a detailed analytical calculation of all possible plasmonic resonances that the Au ring structure can support in the spectral range of 500–2000 nm (0.6–2.5 eV) (a quasi-normal-mode expansion formalism^49^ was used; see Materials and methods for details). A comparison of the results is presented in Fig. S1. Additionally, there are two multipolar dark modes with energies between those of the antibonding and bonding modes. These modes are the ***S***_***6***_ mode at ~775 nm (~1.6 eV) and the quadrupolar ***S***_***4***_ mode at longer wavelengths, slightly above 1000 nm (~1.2 eV). The EELS spectrum shown in Fig. S1b, which is an average of spectra taken on different Au-RIs to account for the size distribution and nanofabrication defects, displays 4 clear peaks, one broad and centred at 2.1 eV (590 nm, antibonding mode) and three narrower peaks centred at 1.55 eV (800 nm, ***S***_***6***_ mode), 1.2 eV (1050 nm, ***S***_***4***_ quadrupolar mode), and 0.72 eV (1720 nm, dipolar bonding mode), in excellent agreement with the predictions of our numerical simulations and analytical calculations.

In our NCRD nanocavity, coupling of the dipolar plasmon resonance of the Py disk nanoantenna can occur only with the strong bright antibonding mode and with the ***S***_***6***_ dark mode at wavelengths of approximately 600 nm and 800 nm, respectively. We, therefore, expect to see only three features in the spectrum of NCRD structures at wavelengths of approximately 600 nm, 800 nm, and above 1600 nm, which is exactly what we observe in the simulated and measured spectra (Fig. 2a, b).

**Fig. 3:**
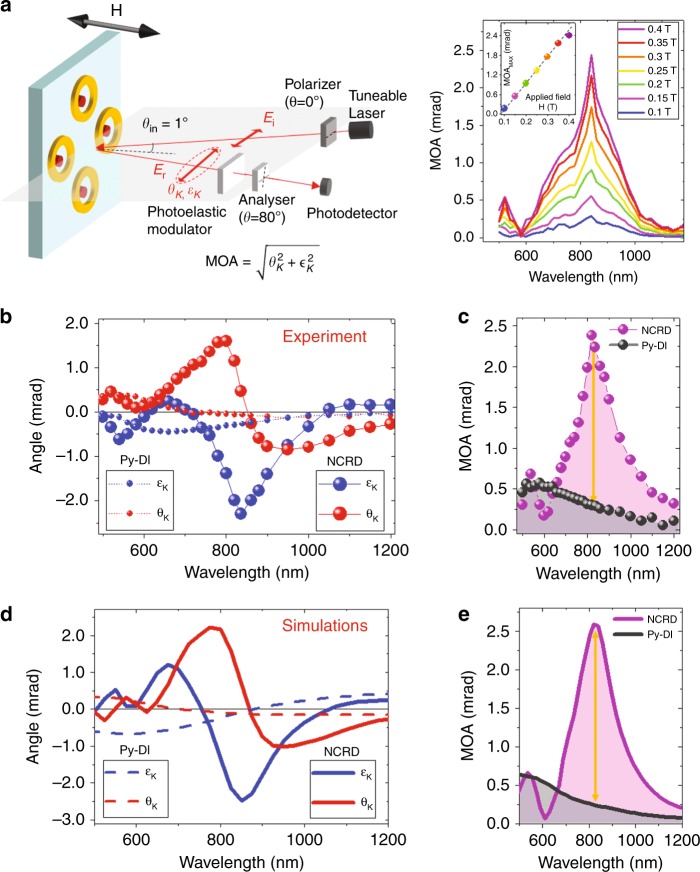
Magneto-optical response of the NCRD hybrid structure and the bare Py nanoantenna. **a** Schematic of the magneto-optical Kerr effect spectrometer. The setup consists of a broadband supercontinuum tuneable laser, polarizing and focusing optics, a photoelastic modulator, and a photodetector. The instrument is operated under nearly normal incidence (incidence angle of 1°) with linearly polarized light in the scattering plane (shadowed plane in the figure). The sample is rotated so that the narrow gap between the Py disk and the Au ring lies in the scattering plane (i.e., parallel to the electric field of the incident radiation). An out-of-plane magnetic field from an electromagnet saturates the magnetization of the Py nanodisks. The left subpanel shows the magnetic field dependence of the magneto-optical activity (MOA) spectral dependence, i.e., of the total polarization swing, upon switching the sign of the applied magnetic field ***H***. d is the applied magnetic field dependence of the maximum induced polarization swing. **b** Kerr rotation (θ_K_) and ellipticity (ε_K_) and **c** MOA experimental spectra for NCRD and Py-DI structures. **d** Kerr rotation (θ_K_) and ellipticity (ε_K_) and **e** MOA simulated spectra for NCRD and Py-DI structures. The dark orange arrows in (**c**, **e**) mark the amplification of the MOA at resonance for the NCRD with respect to Py-DI structures at the same wavelength. The noise level of the measurements is 2μradians (standard deviation), namely, ~2 orders of magnitude smaller than the smallest signal measured in the experiments. Therefore, the error bars are far smaller than the size of the symbols used in the plots of (**b**, **c**)

Once we attained a clear overview of the main optical features, we then investigated the physics of the hybridization of bright and dark modes in the magnetoplasmonic nanocavity and its effects on the ***H***-induced light polarization modulation in the relevant spectral range, i.e., from 500 nm to 1200 nm, where the hybridization should occur. The study was performed by measuring MO Kerr effect (MOKE) spectra, namely, the polarization rotation (θ_K_) and ellipticity (ε_K_), of reflected light while changing the magnetization of the Py disk, with ***H*** applied perpendicular to the sample plane. Figure 3a shows a schematic of the MOKE configuration utilized in the experiment (polar MOKE configuration; see Materials and methods for details). As shown in Fig. 3a, the array is rotated by 45° so that the narrow gap between the Py disk and the Au ring lies in the scattering plane (i.e., parallel to the electric field of the incident radiation). For this experimental geometry, the possible excitation of an SLR via diffractive coupling through air should occur at a wavelength of 1100 nm. Therefore, although we already discussed the fact that effects arising from the excitation of SLRs are negligibly small in the optical response (see discussion of Fig. 2a, b above), any possible influence of the SLRs on the MOKE data can be ruled out completely in the spectral range 500–1100 nm. From the spectra of θ_K_ and ε_K_, the spectral dependence of the ***H***-induced modulation of light polarization is numerically quantified by the modulus |Θ_K_| of the complex Kerr angle, Θ_K_ = (θ_K_ + i ε_K_). This quantity is conventionally named MO activity, hereafter referred to as MOA. The measured θ_K_, ε_K_, and MOA spectra are reported in Fig. 3b, c for both the Py-DI and NCRD structures.

A clear difference can be observed in both the shape and intensity of the experimental MOA for the Py-DI and NCRD structures (Fig. 3c). For the case of the Py-DI nanoantennas, the MOA spectrum displays the usual features of magnetoplasmonic nanostructures, with a maximum of the MOA (gray balls in Fig. 3c) in the spectral range where the dipolar LPR is observed, with the characteristic oscillating behavior of θ_K_ and ε_K_ (solid dots in Fig. 3b). These lineshapes are well understood and known to arise from the interplay between the amplitude and phase of the LPR and MO-LPR. The fact that MOKE signals of the Py-DI sample can be clearly measured in the spectral range where the LPR is excited (500–900 nm) despite the fact that the nanoantennas cover only a minute fraction (≈1.2%) of the sample surface is the result of the well-studied bright plasmon enhancement of the MOA. It is worth noting here that for NCRD structures, only the Py disk contributes to the MOKE signal, as any ***H***-dependent contribution from the Au-ring portion of the nanocavity is not measurable for the weak ***H*** utilized in the experiment (indeed, the Au-RI sample does not show any detectable MOKE signal). Therefore, the surface density of the magneto-optical active material is exactly the same in both the Py-DI and NCRD samples, and the MOKE spectra can be compared side by side. The most striking result shown in Fig. 3b, c is the additional sevenfold enhancement of the MOA, θ_K_, and ε_K_ on the NCRD nanocavities near a wavelength of 820 nm with respect to the Py-DI nanoantennas (marked by the orange arrows in Fig. 3c). The highly enhanced MOA at 820 nm can also be appreciated by looking at the direct comparison of the measured θ_K_ and ε_K_ signals at 820 nm as a function of the applied field ***H*** for the NCRD and Py-DI shown in Fig. S2. In addition, in Fig. 3a (right-hand panel), we report the spectral dependence of the MOA, i.e., the polarization swing, upon switching the sign of an applied magnetic field ***H*** of different intensities. The inset of Fig. 3a (right panel) shows the applied magnetic field dependence of the maximum induced polarization swing at 820 nm. Figure 3a shows that our structures enable a dynamic and linear tunability of the MO response. The experimental spectral lineshapes of the MOA, θ_K_, and ε_K_ are excellently reproduced by simulations, as can be seen in the comparison of Fig. 3b, c with Fig. 3d, e. The simulations in Fig. 3d predict a slightly larger enhancement (~10% higher) of the MOA, θ_K_, and ε_K_ for defect-free NCRD nanocavities. This small discrepancy is explainable considering that we used tabulated values of the dielectric and magneto-optical constants of Py (the real values are normally slightly different), considering the size distribution of the gap width, and considering the other imperfections affecting the real structures, resulting in a deviation from the ideal shapes utilized in the simulations. Regarding the gap width, since this is a key parameter in the design of a nanocavity, we conducted systematic simulations where the gap width was reduced and increased by 5 nm with respect to the value of 10 nm of our structures. The results are shown in Fig. S3. The MOA decreases as the gap width increases as a consequence of the progressive weakening of the near-field coupling between the dipolar mode of the disk and the multipolar mode of the ring, namely, the hybridization strength. It is therefore clear that further reducing the gap width below the present value of 10 nm would improve the MO enhancement, although not dramatically. The nanolithography of metals is ultimately limited by the grain size of the material, which is typically on the order of 5 nm, depending on the metal and the growth method and conditions. Therefore, the margin for improving the MOA by reducing the gap width is somehow limited.

For the experimental geometry utilized to record the MOKE spectra as well as for the simulations shown in Fig. 3, the possible excitation of an SLR via diffractive coupling through air should occur at a wavelength of 1100 nm (through the glass substrate, the excitation of the SLR is expected at 1680 nm). Therefore, any possible influence of an SLR enhancement on the MOA enhancement observed at 820 nm can be ruled out completely.

## Discussion

**Fig. 4:**
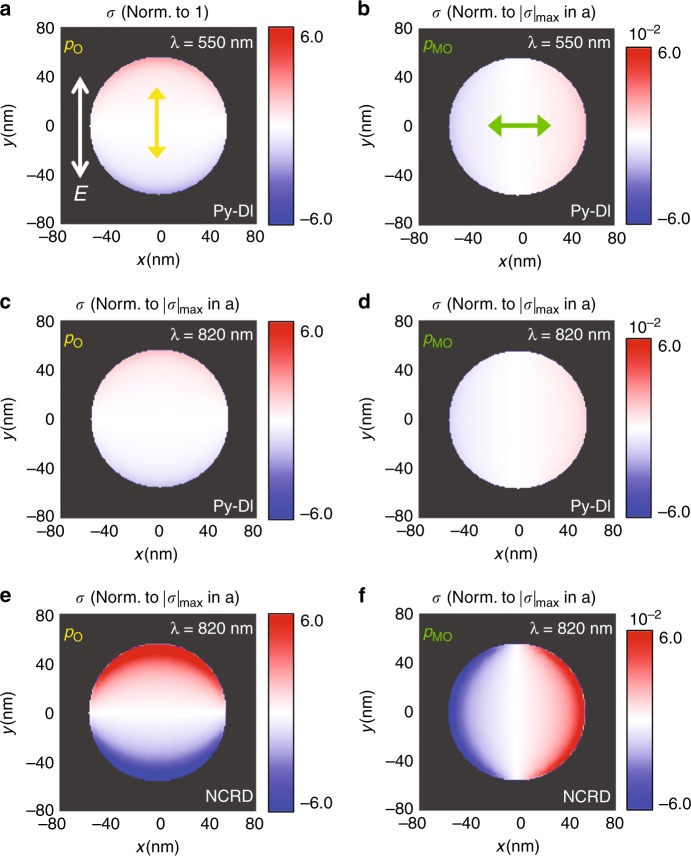
Relative strength of the induced optical (*p*_0_) and magneto-optical (*p*_*MO*_) electric dipoles of the Py disk in the NCRD structure and a standalone Py disk. 2D maps of the calculated surface charge distribution *σ* at the interface between the structures and the substrate. The intensity of all maps is normalized to the maximum value of |*σ*| (|*σ*|_max_) produced by the optical dipole *p*_*O*_ for the Py-DI at 550 nm shown in (**a**). The yellow arrow in a) displays the direction of the optical dipole *p*_*O*_. **b** displays the 2D map of normalized *σ* produced by the magnetic field (***H***)-induced electric dipole *p*_*MO*_ (the green arrow shows its direction) at the same wavelength of 550 nm (details of the calculation given in Materials and methods). **c**, **d** Display the corresponding 2D maps of the normalized *σ* for the Py-DI at a wavelength of 820 nm. **e**, **f** Show the corresponding 2D maps of the normalized *σ* for the NCRD at a wavelength of 820 nm (resonance maximum). *E*_*i*_ in (**a**) (equal to 1 V/m for all maps) shows the direction along which the incident light is polarized. The color scale range of the 2D maps in each column is kept fixed to better highlight the relative sizes of the induced electric dipoles. The range in each column is chosen as the ratio between |*σ*|_max_ in the NCRD at 820nm and that in the Py-DI at 550 nm corresponding to *p*_*O*_ (first column) and *p*_*MO*_ (second column). The σ maps at 820 nm for the Py nanodisk inside the NCRD showed the appearance of a weak quadrupolar mode (***S***_*4*_) superimposed on the intense and dominating dipolar modes ***p***_***O***_ and ***p***_***MO***_ (caused by the hybridization of ***p***_***O***_ with mode ***S***_***6***_ of the nanoring; see Fig. S4 and its caption and Supplementary Video_V1). Since this weak quadrupolar mode is non-radiative and thus not relevant to this discussion, its contribution was removed from the figure to simplify the relative comparison of the induced dipole strengths (see discussion in the caption of Fig. S4)

To shed light on the underlying mechanism at work in the case of NCRD nanocavities at a wavelength of 820 nm, we first calculated the relative strength of the photoinduced electric dipole *p*_*O*_ associated with the LPR and of the ***H***-activated electric dipole *p*_*MO*_ associated with the MO-LPR both in the Py-DI and in the Py nanodisk as part of an NCRD nanocavity. The results of this comparative analysis are summarized in Fig. 4, which displays the computed 2D maps of the surface charge (*σ*) generated by *p*_*O*_ and *p*_*MO*_ (see Materials and methods and Fig. S4 for details). The most interesting result from the comparison of the maps in Fig. 4 is that at 820 nm, the strength of both induced dipoles *p*_*O*_ and *p*_*MO*_, which is proportional to the maximum value of |σ| (|σ|_max_), in the Py disk inside the NCRD nanocavity is much larger (~1 order of magnitude at 820 nm) than that in the bare Py-DI nanoantenna at the same wavelength and even at full resonance at 550 nm.

**Fig. 5:**
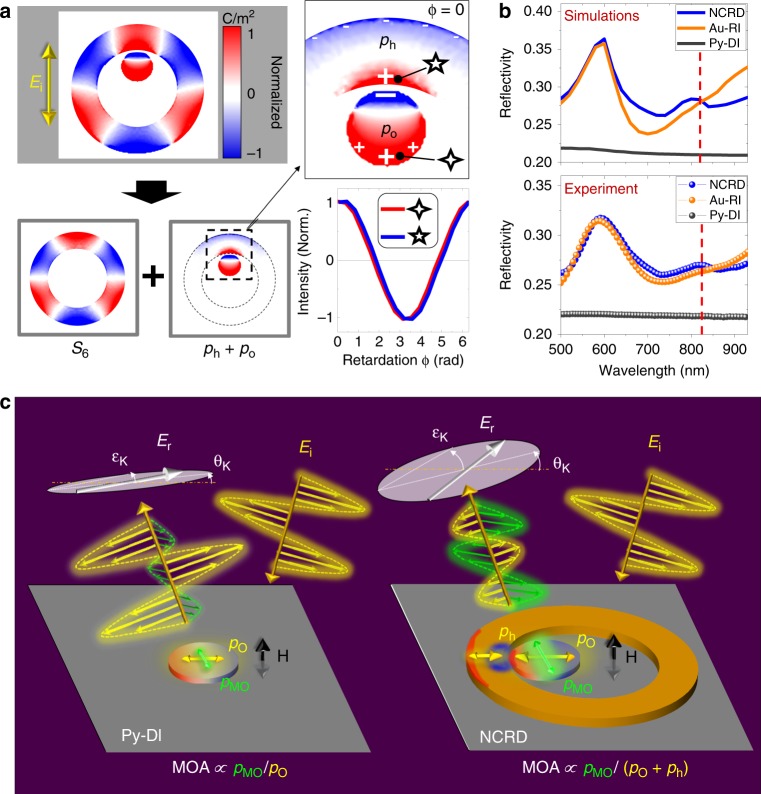
Illustration of the resonant mechanism leading to the large magneto-optical enhancement in the NCRD nanocavity at the Fano resonance maximum. **a** Separation of the hybrid mode into its dipolar (***p***_***O***_ and ***p***_***h***_) and multipolar (***S***_***6***_) components (these are the surface charge maps corresponding to phase ϕ_0_ in the first column of Fig. S3). The panel also shows the time evolution (retardation ϕ − ϕ_0_) of the surface charge at the points marked by the 4- and 5-pointed stars. **b** Calculated and measured optical reflectivity spectra for the NCRD, Au-RI and Py-DI structures. Red dashed lines mark the wavelength of 820 nm, showing that the reflectivity of the NCRD is only marginally higher than that of the Au-RI. The reflectivities of both the NCRD and Au-RI are approximately 25% higher than that of the Py-DI. **c** A sketch of the electrodynamics of the Py disk generating an electric dipole (***p***_***O***_) triggered by the electric field ***E***_***i***_ of an incident linearly polarized electromagnetic radiation and a magneto-optically activated electric dipole (***p***_***MO***_) by a magnetic field ***H***. ***p***_***O***_ and ***p***_***MO***_ of the Py nanoantenna inside the NCRD nanocavity are enhanced (by a factor of ~5) with respect to a bare Py disk (Py-DI) by hybridization with the mode ***S***_***6***_ of the Au-RI, which results in the introduction of a dipolar mode, ***p***_***h***_, in the ***S***_***6***_ mode. This is qualitatively depicted by the relative size of electric dipoles ***p***_***O***_ and ***p***_***MO***_ in the Py-DI and in the NCRD. In the NCRD nanocavity, hybridization generates a bonding dipolar mode ***p***_***O***_ + ***p***_***h***_. In the Py-DI system, both ***p***_***O***_ and ***p***_***MO***_ are generated by *radiant* (bright) LPR modes, and the resulting ***H-***induced polarization change in the reflected radiation, ***E***_***r***_, is determined by their ratio (MOA ∝ |***p***_***MO***_|/|***p***_***O***_|, θ_K_ ∝ Re[***p***_***MO***_/***p***_***O***_], and ε_K_ ∝ Im[***p***_***MO***_/***p***_***O***_]). The large enhancement of the ***H***-induced polarization change in the NCRD system is a consequence of the low-radiant character of the bonding dipolar mode (***p***_***O*** _+ ***p***_***h***_), while ***p***_***MO***_ maintains its enhancement and *radiant* character

This remarkable enhancement occurs even though the Py disk is not driven at full resonance (the strength of *p*_*O*_ and *p*_*MO*_ is reduced by 20% at 820 nm, as we evaluated by comparing Fig. 4a with Fig. 4c or, equivalently, Fig. 4b with Fig. 4d). The explanation for this prominent enhancement comes from the detailed analysis of the plasmonic coupling-induced electrodynamics in the Py disk inside the NCRD nanocavity. This is performed by simulated comparative monitoring of the time evolution of the charge density induced by the electric field of the incident light in the NCRD cavity and standalone Au-RI. At each time, the surface charge map of the Au-RI (multiplied by a factor, see Fig. 2c) is subtracted from that of the NCRD nanocavity. This allows the identification of the modes (and their symmetry) that hybridize in the NCRD nanocavity. This complete modal analysis is shown in Fig. S5, and the key results are summarized in Fig. 5a.

Figure 5a (as well as Fig. S5) clearly shows that the electrodynamics of the NCRD at 820 nm (see also Supplementary Video_V1) is determined by the resonance resulting from hybridization between the bright dipolar mode ***p***_***O***_ of the Py nanodisk with the ***S***_***6***_ dark multipolar mode of the Au ring, forming a nanocavity hybrid multipolar mode referred to as multipolar Fano or octupolar Fano resonance in the literature. The electrodynamics originating from the strong and localized near-field coupling between the two parent modes corresponds to the introduction of an additional localized dipolar mode, ***p***_***h***_, into the ring nanocavity (marked on Fig. 5a). Therefore, overall, at the multipolar resonance, the electrodynamics (maps in the top line of Fig. S5) results from the interference of a coupled mode ***p***_***c***_ = ***p***_***O***_ + ***p***_***h***_ (maps in the bottom line of Fig. S5) and the dark multipolar ***S***_***6***_ mode (maps in the middle line of Figure S5). The analysis of the relative phase of the electric dipoles *p*_*h*_ and *p*_*O*_ associated with the coupled mode ***p***_***c***_ reveals that at the wavelength of 820 nm, the two dipoles oscillate in phase. Therefore, at this wavelength, the resonance displays its maximum due to constructive interference. In this situation, the coupled mode ***p***_*c*_ has a bonding character, which results in the marked enhancement of the local dipole *p*_*O*_ of the Py disk. This enhancement is transferred to the ***H***-activated dipolar mode ***p***_***MO***_ via MO coupling in Py (compare Fig. 4b, f). Notably, simulations also show that the ***p***_***MO***_ mode is a bright mode, as it does not show significant direct hybridization with any mode of the ring nanoresonator.

**Fig. 6:**
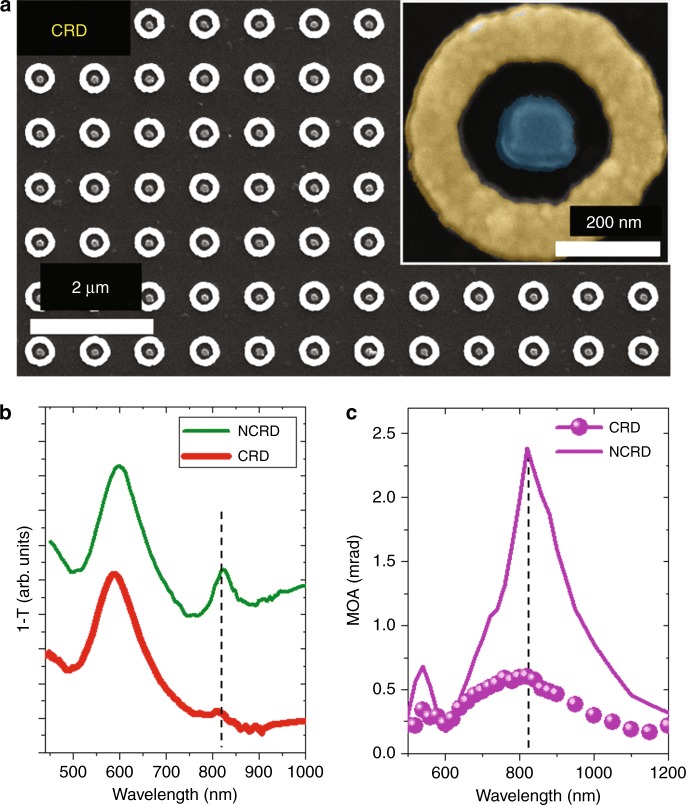
SEM and MOKE of the CDR cavities. **a** Scanning electron microscopy images of the array of CRD nanocavities. **b** Comparison between the experimental transmittance spectra for the CRD and NCRD nanocavities. **c** Comparison between the MOA experimental spectra for the CRD (solid symbols), NCRD (purple line), and Py-DI (gray line) structures. The noise level of the MO measurements is 2 μradians (standard deviation), namely, ~2 orders of magnitude smaller than the smallest signal measured in the experiments. Therefore, the error bars are far smaller than the size of the symbols and the line used in the plots of (**c**)

Although intriguing, per se, the simultaneous enhancement of the electric dipoles *p*_*O*_ and *p*_*MO*_ does not explain the surprisingly large enhancement of the MOA produced by the NCRD nanocavity at 820 nm. Indeed, if both dipoles were bright, i.e., radiant, the MOA would be again Q-fold limited and quite similar for the three cases analysed in Fig. 4. The answer to our puzzle is again contained in Fig. 5a and Fig. S5: the uneven charge distribution in the bonding-coupled ***p***_***c***_ mode (dominating tri-polar character; see Fig. 5a) and its interference with the distorted ***S***_***6***_ mode make the resulting hybrid multipolar Fano resonance of low radiance. The low-radiant character of the Fano resonance mode at 820 nm is confirmed by Fig. 5b, where we plot the calculated and measured reflectivity spectra *E*_*r*_/*E*_*i*_, with *E*_*i*_ and *E*_*r*_ being the incident and reflected electric far fields. Figure 5b clearly shows that the excitation of the multipolar Fano resonance mode enhances the reflectivity of the NCRD only marginally with respect to the Au-RI. Figure 5b also shows that the reflectivity of NCRD at 820 nm is only 25% larger than that of Py-DI (see also Fig. S6a, which features the ratio between the reflectivity of the NCRD and Py-DI). This indicates that the dip at a wavelength of approximately 820 nm in the calculated and measured transmittance spectra (Fig. 2a, b) is predominantly due to light absorption, as illustrated by the comparison between the absorption cross section of the NCRD and Au-RI in Fig. S6b and, as expected, from a low-radiant mode. The physical mechanism behind the large MOA amplification achieved in the NCRD, much larger than that of the already enhanced parent magnetoplasmonic Py-DI structure, is now clear and is summarized in pictorial form in the images shown in Fig. 5c. The images depict schematically how, at a wavelength of 820 nm, the hybridization between ***p***_**O**_ and ***S***_***6***_ in the NCRD nanocavity leads to excitation of the low-radiant bonding mode ***p***_***c***_ (the ***S***_***6***_ mode of the Fano resonance is not shown in Fig. 5a for clarity), while the ***H***-activated bright dipolar mode ***p***_***MO***_, which only weakly interacts with the Au ring, inherits the large enhancement from ***p***_***O***_ while preserving its *radiant* character. As a consequence, the unique electrodynamics produced by the hybridization in the nanocavity occurring at ~820 nm generates a strongly enhanced ***H***-activated bright electric dipole *p*_*MO*_ without paying the price of a parallel increase in the re-emitted radiation with the primary polarization, thanks to the low-radiant character of the driving hybrid multipolar Fano mode. The result is a large MOA amplification, beyond the Q-fold limit in the NCRD nanocavity with respect to the parent and Q-fold limited magnetoplasmonic Py-DI structure (Fig. 3c, e). Further corroborating the uniqueness of the electrodynamics occurring in the NCRD nanocavity at 820 nm (see Supplementary Video_V1), we note that even stronger hybridization of the LPR of the Py disk with the intense antibonding mode of the Au ring in the spectral range of 500–600 nm (Fig. 2c) does not produce any enhancement in the MOA of the NCRD with respect to the Py-DI. Remarkably, it is rather the opposite: the large enhancement of the re-emitted radiation by this radiant mode in the Au ring results in a weakening of the MO response at 600 nm, as exhibited by the spectra in Fig. 3c, e. We also performed a study of the dependence of the NCRD optical and MOA spectra on the angle of polarization of the incident light, which are reported in Fig. S7. The comparison of the spectra confirms that the polarization angle chosen for the experimental results reported in Fig. 3 is the one that maximizes the coupling between the bright dipolar mode of the disk and the multipolar dark mode of the ring and leads to the highest MOA amplification (angle identified with −45° in Fig. S7). At other angles of polarization, the coupling between the modes of the disk and the ring becomes weaker, and the MOA enhancement, although preserving the spectral lineshape, is slightly reduced as a consequence of the weaker coupling. These results further corroborate the correctness of our understanding of the physical mechanism responsible for the large MOA enhancement, as described in the manuscript. Finally, and as a further proof, we report in Fig. 6 the measured optical (Fig. 6b) and MO (Fig. 6c) responses of a concentric ring/disk (CRD) nanocavity with the sizes of the constituent parts nominally identical to those of the NCRD (see the SEM image in Fig. 6a). For this CRD nanocavity, the MO response at 820 nm should be the same as that of the Py-DI structures (see surface charge maps in Fig. S8b) given the geometrical symmetry of the structure. The experimental MOA of the CRD sample is indeed only weakly modified with respect to the Py-DI, as appreciable in Fig. 6c, because of the non-perfect concentricity of the nanocavity.

In conclusion, we have demonstrated that high-order multipolar dark plasmon resonances in magnetoplasmonic nanocavities can be utilized to achieve unprecedented enhancement of the magneto-activated optical response, beyond the present limitations of magnetoplasmonic nanoantennas, enabling far more efficient active control of the light polarization under weak magnetic fields. The superior behavior of geometrical symmetry-broken magnetoplasmonic nanocavities compared to that of the corresponding nanoantennas is explained by the generation of a largely enhanced magnetic-field-induced radiant dipole in the magnetoplasmonic nanoantenna driven by a hybrid low-radiant multipolar Fano resonance mode. Therefore, in this novel design, a large enhancement of the magneto-optical response, i.e., a magneto-activated electrical dipole inducing modification of the light polarization, is achieved without a significant increase in the pure optical response thanks to the low-radiant character of the hybrid mode. As a result, in the NCRD magnetoplasmonic nanocavity, the MOA is amplified well beyond that of the parent Py-DI structure. The novel concept unveiled here opens up a new path towards applications of magnetoplasmonics to a variety of fields ranging from flat and active nanophotonics to sensing. An improved design of the cavities along with a proper selection of the materials with higher a Q-factor and more intrinsic MO activity than those of Permalloy will allow achieving unprecedented values of polarization modulation for magnetically controlled nanophotonic devices. One direction to explore is the tuning of the resonance frequencies of dark and bright plasmon modes to bring them closer. This can be achieved by using silver instead of gold for the nanoring to produce a blue-shift of the multipolar resonance and utilizing multilayer gold/cobalt for the MO-active nanoantenna. The utilization of a gold/cobalt multilayered nanoantenna will produce a red-shift of the dipolar resonance required to strengthen hybridization and will increase the Q-factor of the dipolar resonance and the intrinsic MO activity of the nanoantenna. As discussed in the manuscript, the strength of the hybridization can also be increased by reducing the gap between the nanodisk and the ring, although the margin for improvement is somehow limited by the relatively large grain size (~5 nm). Obviously, the other parameter that needs to be improved is the surface coverage, which can be done by reducing the footprint of the nanocavity (in our demonstrative design, the surface coverage of the MO-active material is only 1.2%). Using the nanocavity design described here, one can envision that an improvement of 2–3 times is within reach based on the present state-of-the-art nanolithography. Further improvements in this direction require a change in the design. For example, multipolar modes can be excited by near-field coupling in more compact structures such as vertical hetero-structured dimers (e.g., gold/silicon-oxide/cobalt nanoantennas)^39^. The implementation of nanocavities made of heterogeneous multimeric structures (trimers, tetramers, etc.) should also be explored as a way to realize high-Q nanocavities with a reduced footprint. Reducing the footprint of the nanocavity unit would allow the realization of metagratings, where the interference effect produced by light re-emitted by the individual units when the gratings are illuminated at oblique incidence can be exploited to achieve large polarization changes in selected diffraction orders^50–52^. The utilization of a dielectric ring instead of a gold ring should also be considered since it could result in a substantial increase in the Q-factor of the hybrid multipolar resonance. Along the line of enhancing the Q-factor, the excitation of surface lattice resonances can be exploited by reducing the array pitch and embedding the array in a homogeneous medium (e.g., embedding the array in silicon oxide).

In conclusion, the underlying mechanism leading to the enhanced MOA presented in this work suggests a multitude of directions to be explored to advance beyond this proof-of-principle study.

## Materials and methods

### Fabrication

The samples were fabricated by electron-beam (e-beam) lithography and the lift-off procedure. Initially, a 5-nm-thick Ti layer was e-beam evaporated (evaporation rate: 0.4 Å/s) onto a cleaned 10 mm × 10 mm Pyrex substrate as an adhesion layer and as a metallic coating to avoid charging effects during e-beam lithography. The NCRD structures were grown by a sequential two-step procedure. First, the Py disk was prepared by spin-coating a positive resist (ZEP520A-7) onto the substrate at 4000 rpm for 60 s. The resist was exposed by a 20-KV electron beam inside a RAITH eLine system. The exposition time was adjusted according to the dot size. After developing the exposed resist (with the ZED-N50 developer), a 40-nm-thick Py layer was thermally evaporated (evaporation rate: 0.8 Å/s). Finally, lift-off was carried out by dipping the samples into the proper solvent (ZDMAC). Eventually, the process was repeated to fabricate the Au ring around the disks. The process was then repeated to fabricate the Au ring around the Py disks, taking special care in the electron beam optimization (beam focusing, aperture alignment and astigmatism correction) and in the second step of the alignment procedure.

### Structural, optic, electron, and atomic force microscopy and magneto-optic characterization

Scanning electron microscopy images were recorded by an eSEM-FEI QuantaTM 250 instrument operating at an accelerating voltage of 10 kV. The transmittance spectra were taken in the wavelength range of 400–1600 nm using Pyrex glass as the background signal. Electron energy loss spectra (EELS) and scanning transmission electron microscopy (STEM) images were acquired using a TitanG2 60-300 (FEI, Netherlands) operating at 80 kV in monochromatic mode (energy resolution of ~80 meV). Atomic force microscopy images were acquired in air under ambient conditions using a Nano Observer system (CSI Company, France). Measurements were made in tapping mode using an NCHV-A (Bruker) tip with a spring constant k = 40 N/m and a resonance frequency of 320 kHz. Magneto-optical Kerr effect measurements were conducted with a Kerr spectrometer. A schematic of the setup and geometry utilized in the experiment is shown in the left panel of Fig. 3a. The setup consisted of a broadband supercontinuum laser (SuperK Extreme EXR-15 from NKT Photonics), polarizing and focusing optics, a photoelastic modulator (Hinds Instruments II/FS42A), and a Si-photodetector (Thorlabs PDA 36A-EC). The wavelength of the laser was tuned between 500 and 1200 nm. We used linear polarized light impinging on the sample at near-to-normal incidence (polar Kerr configuration; the angle of incidence was 1° with respect to the sample normal). The polar Kerr configuration was selected since both optical- and magneto-optical-induced dipoles can be resonantly excited in the sample plain, a situation that is the most favorable for flat structures such as those studied here. In the longitudinal and transverse Kerr configurations, one of these two dipoles will be perpendicular to the sample surface, and to have it resonantly induced at optical frequencies, the height of the ferromagnetic nanostructure should be at least equal to its diameter (see ref. ^9^), i.e., 100 nm or higher, making nanofabrication extremely challenging. During measurements, a ±700-mT magnetic field from an electromagnet switched the magnetization of the Py nanodisks between the two perpendicular directions (the field for magnetic saturation is approximately ±400 mT; see Fig. S2). Two lock-in amplifiers were used to filter the signal at the modulation frequency (42 kHz) and at twice the modulation frequency to retrieve the Kerr ellipticity (ε_K_) and rotation (θ_K_) angles simultaneously^53^. The data shown in Fig. 3 and Fig. S5 refer to [θ_K_(+H) – θ_K_(−H)], [ε_K_(+H) – ε_K_(−H)], and [|Θ_K_ |(+ H) – |Θ_K_|(−H)], where Θ_K_ = (θ_K_ + i ε_K_). The limit of detection of our MOKE setup, i.e., the noise level, is 2 μradians (standard deviation), which is ~2 orders of magnitude smaller than the smallest signal measured in the reported experiments. Therefore, the error bars are smaller than the size of the symbols utilized in the plots (Fig. 3b, c, S2c and S2d).

### Simulations

#### **Electromagnetic simulations**

3D electrodynamic calculations of the optical transmittance and the surface charge density maps were performed by solving the Maxwell equations via the finite element method implemented in the commercial COMSOL Multiphysics software^54^ using the RF module in the frequency domain. The experimental structures (Py-DI, Au-RI, and NCRD) were modeled as arrays using the standard port formulation and periodic boundary conditions. Hence, the physical domains were placed in regular square array arrangements with a pitch of 800 nm along both in-plane axes and influenced by linearly polarized light at normal incidence. We used air, n_air_ = 1.0, for the incoming light environment, a substrate with a refractive index *n* = 1.5 (mimicking Pyrex) and Au dielectric optical functions from Johnson and Christy^55^. For the magneto-plasmonic structure (Py-DI), we consider a non-diagonal dielectric tensor medium in which the non-zero off-diagonal elements depend on the applied magnetic field, the orientation of the geometry and the polarization of the incoming light. For our case, i.e., light reflected through the sample with an applied magnetic field perpendicular to the surface of the sample (polar Kerr configuration), the dielectric tensor for the Py-DI in terms of the diagonal ε_*d*_(*ω*) and off-diagonal ε_*od*_(*ω*) terms adopts the form

$${\upvarepsilon}\left( \omega \right) = \left( {\begin{array}{*{20}{c}} {{\upvarepsilon}_d(\omega )} & { \mp {\upvarepsilon}_{od}(\omega )} & 0 \\ { \pm {\upvarepsilon}_{od}(\omega )} & {{\upvarepsilon}_d(\omega )} & 0 \\ 0 & 0 & {{\upvarepsilon}_d(\omega )} \end{array}} \right),$$

where ε_*d*_(*ω*) and ε_*od*_(ω) for Ni are taken from ref. ^56^. We used the dielectric properties of Ni as representative of Py since the two materials have almost identical optical and magneto-optical properties and the dielectric tensor constants of the former are available over a larger spectral range. The inversion of the sign in the off-diagonal elements mimics the effect of the reversal of the static magnetic field from +*H* to −*H*. All domains were meshed by using tetrahedral elements for which the maximum mesh element size was kept below λ/10, where λ is the wavelength of the incident light. For the elements corresponding to both the ring and the disk domains, the size was ten times finer than the largest element size (verified to properly resolve the considered structures).

The 2D maps of the surface charge density (Figs. 2c, 5a, Figs. S3, S4, and S6d) used for the relative comparison of the dipole strengths were obtained by plotting computed σ = **P·n** in a plane parallel to the interface between the structures and the substrate (**P** is the electric dipole per unit surface, i.e., the electric polarization, and **n** is the normal to the surface). To extract the 2D maps of σ produced by the MO-LPR in Fig. 4 and of |σ| in Fig, S4d, we subtracted the COMSOL-calculated **P·n** at *+****H*** and −***H***. In this respect, we mention here that the σ maps at 820 nm for the Py nanodisk for the NCRD showed the appearance of a weak quadrupolar mode (***S***_***4***_) superimposed onto the intense and dominating dipolar modes ***p***_***O***_ and ***p***_***MO***_ (caused by hybridization with mode ***S***_***6***_ of the nanoring). Since this weak quadrupolar mode is non-radiative and thus not relevant to this discussion, its contribution was removed to simplify the relative comparison of induced dipole strengths.

### Normal mode simulations

For the calculation of the Au-RI plasmonic normal modes, we adapted the geometry of our system to the efficient finite-element solver (QNMEig) developed in COMSOL Multiphysics by Yan et al.^49^, which uses a quasi-normal mode (QNM)-expansion formalism to compute the resonance modes of an absorptive and dispersive plasmonic nanoresonator, solving a standard linear eigenvalue problem derived from Maxwell’s equations. A key quantity retrieved by the formalism is the electric polarization **P**, from which the surface charge density can be calculated.

## Supplementary information

Supplementary Information

Supplementary Vide V1

## Data Availability

The authors declare that all data supporting the findings of this study are available within the paper and its Supplementary Information files.

## References

[1] Koenderink AF, Alù A, Polman A (2015). Nanophotonics: shrinking light-based technology. Science.

[2] Northup TE, Blatt R (2014). Quantum information transfer using photons. Nat. Photonics.

[3] Joannopoulos JD, Villeneuve PR, Fan S (1997). Photonic crystals: putting a new twist on light. Nature.

[4] Shelby RA, Smith DR, Schultz S (2001). Experimental verification of a negative index of refraction. Science.

[5] Maccaferri N (2015). Resonant enhancement of magneto-optical activity induced by surface plasmon polariton modes coupling in 2D magnetoplasmonic crystals. ACS Photonics.

[6] Chen J (2011). Plasmonic nickel nanoantennas. Small.

[7] Bonanni V (2011). Designer magnetoplasmonics with nickel nanoferromagnets. Nano Lett..

[8] Lodewijks K (2014). Magnetoplasmonic design rules for active magneto-optics. Nano Lett..

[9] Berger, A. et al. Enhanced magneto-optical edge excitation in nanoscale magnetic disks. *Phys. Rev. Lett*. **115**, 187403 (2015). 10.1103/PhysRevLett.115.187403 26565496

[10] Maccaferri N (2016). Anisotropic nanoantenna-based magnetoplasmonic crystals for highly enhanced and tunable magneto-optical activity. Nano Lett..

[11] Maccaferri N (2015). Ultrasensitive and label-free molecular-level detection enabled by light phase control in magnetoplasmonic nanoantennas. Nat. Commun..

[12] Valev VK (2011). Plasmons reveal the direction of magnetization in nickel nanostructures. ACS Nano.

[13] González-Díaz JB (2008). Plasmonic Au/Co/Au Nanosandwiches with Enhanced Magneto-optical Activity. Small.

[14] Banthí, J. C. et al. High magneto-optical activity and low optical losses in metal-dielectric Au/Co/Au-SiO2 magnetoplasmonic nanodisks. *Adv Mater* **24**, OP36–OP41 (2012). 10.1002/adma.201103634 22213149

[15] Chin JY (2013). Nonreciprocal plasmonics enables giant enhancement of thin-film Faraday rotation. Nat. Commun..

[16] Belotelov VI (2011). Enhanced magneto-optical effects in magnetoplasmonic crystals. Nat. Nanotechnol..

[17] Wang L (2011). Plasmonics and enhanced magneto-optics in core−shell Co−Ag nanoparticles. Nano Lett..

[18] López-Ortega A, Takahashi M, Maenosono S, Vavassori P (2018). Plasmon induced magneto-optical enhancement in metallic Ag/FeCo core/shell nanoparticles synthesized by colloidal chemistry. Nanoscale.

[19] Armelles G, Cebollada A, García-Martín A, González MU (2013). Magnetoplasmonics: combining magnetic and plasmonic functionalities. Adv. Opt. Mater..

[20] Maksymov IS (2016). Magneto-plasmonic nanoantennas: basics and applications. Rev. Phys..

[21] Floess D, Weiss T, Tikhodeev S, Giessen H (2016). Lorentz nonreciprocal model for hybrid magnetoplasmonics. Phys. Rev. Lett..

[22] Floess D (2017). Plasmonic analog of electromagnetically induced absorption leads to giant thin film faraday rotation of 14°. Phys. Rev. X.

[23] Uchida H, Masuda Y, Fujikawa R, Baryshev AV, Inoue M (2009). Large enhancement of Faraday rotation by localized surface plasmon resonance in Au nanoparticles embedded in Bi:YIG film. J. Magn. Magn. Mater..

[24] Temnov VV (2010). Active magneto-plasmonics in hybrid metal–ferromagnet structures. Nat. Photonics.

[25] Ferreiro-Vila E, García-Martín JM, Cebollada A, Armelles G, González MU (2013). Magnetic modulation of surface plasmon modes in magnetoplasmonic metal-insulator-metal cavities. Opt. Express.

[26] Zubritskaya I, Maccaferri N, Inchausti Ezeiza X, Vavassori P, Dmitriev A (2018). Magnetic control of the chiroptical plasmonic surfaces. Nano Lett..

[27] Armelles G (2015). Interaction effects between magnetic and chiral building blocks: a new route for tunable magneto-chiral plasmonic structures. ACS Photonics.

[28] Armelles G (2014). Magnetic field modulation of chirooptical effects in magnetoplasmonic structures. Nanoscale.

[29] Feng HY, de Dios C, García F, Cebollada A, Armelles G (2017). Analysis and magnetic modulation of chiro-optical properties in anisotropic chiral and magneto-chiral plasmonic systems. Opt. Express.

[30] Zubritskaya I (2015). Active magnetoplasmonic ruler. Nano Lett..

[31] Pourjamal S, Kataja M, Maccaferri N, Vavassori P, van Dijken S (2018). Hybrid Ni/SiO_2_/Au dimer arrays for high-resolution refractive index sensing. Nanophotonics.

[32] Sepúlveda B, Calle A, Lechuga LM, Armelles G (2006). Highly sensitive detection of biomolecules with the magneto-optic surface-plasmon-resonance sensor. Opt. Lett..

[33] Regatos D, Sepúlveda B, Fariña D, Carrascosa LG, Lechuga LM (2011). Suitable combination of noble/ferromagnetic metal multilayers for enhanced magneto-plasmonic biosensing. Opt. Express.

[34] Caballero B, García-Martín A, Cuevas JC (2016). Hybrid magnetoplasmonic crystals boost the performance of nanohole arrays as plasmonic sensors. ACS Photonics.

[35] Ahmadivand A (2017). Rapid detection of infectious envelope proteins by magnetoplasmonic toroidal metasensors. ACS Sens..

[36] Maccaferri N (2013). Tuning the magneto-optical response of nanosize ferromagnetic ni disks using the phase of localized plasmons. Phys. Rev. Lett..

[37] Klar T (1998). Surface-plasmon resonances in single metallic nanoparticles. Phys. Rev. Lett..

[38] Armelles G (2009). Magnetoplasmonic nanostructures: systems supporting both plasmonic and magnetic properties. J. Opt. A Pure Appl Opt..

[39] Armelles G (2013). Mimicking electromagnetically induced transparency in the magneto-optical activity of magnetoplasmonic nanoresonators. Opt. Express.

[40] de Sousa N (2014). Interaction effects on the magneto-optical response of magnetoplasmonic dimers. Phys. Rev. B.

[41] Pourjamal S, Kataja M, Maccaferri N, Vavassori P, van Dijken S (2019). Tunable magnetoplasmonics in lattices of Ni/SiO2/Au dimers. Sci. Rep..

[42] Feng HY (2017). Active magnetoplasmonic split-ring/ring nanoantennas. Nanoscale.

[43] Sonnefraud Y (2010). Experimental realization of subradiant, superradiant, and fano resonances in ring/disk plasmonic nanocavities. ACS Nano.

[44] Hao F (2008). Symmetry breaking in plasmonic nanocavities: subradiant lspr sensing and a tunable fano resonance. Nano Lett..

[45] Hao F, Larsson EM, Ali TA, Sutherland DS, Nordlander P (2008). Shedding light on dark plasmons in gold nanorings. Chem. Phys. Lett..

[46] Large N (2011). Plasmonic properties of gold ring-disk nano-resonators: fine shape details matter. Opt. Express.

[47] Hao F, Nordlander P, Burnett MT, Maier SA (2007). Enhanced tunability and linewidth sharpening of plasmon resonances in hybridized metallic ring/disk nanocavities. Phys. Rev. B.

[48] Chu M-W (2009). Probing bright and dark surface-plasmon modes in individual and coupled noble metal nanoparticles using an electron beam. Nano Lett..

[49] Yan W, Faggiani R, Lalanne P (2018). Rigorous modal analysis of plasmonic nanoresonators. Phys. Rev. B.

[50] Du J (2011). Optical beam steering based on the symmetry of resonant modes of nanoparticles. Phys. Rev. Lett..

[51] Du J, Lin Z, Chui ST, Dong G, Zhang W (2013). Nearly total omnidirectional reflection by a single layer of nanorods. Phys. Rev. Lett..

[52] Ra’di Y, Sounas DL, Alù A (2017). Metagratings: beyond the limits of graded metasurfaces for wave front control. Phys. Rev. Lett..

[53] Vavassori, P. Polarization modulation technique for magneto-optical quantitative vector magnetometry. *Appl. Phys Lett*. **77**, 1605 (2000).

[54] COMSOL. COMSOL Multyphisics v. 5.2, AB, Stockholm, Sweden. http://www.comsol.com.

[55] Johnson PB, Christy RW (1972). Optical constants of the noble metals. Phys. Rev. B.

[56] Višňovský Š (1993). Magneto-optical Kerr spectra of nickel. J. Magn. Magn. Mater..

