# Supplementary material for: Enhanced magnetic modulation of light polarization exploiting hybridization with multipolar dark plasmons in magnetoplasmonic nanocavities
Source: Light Sci Appl. 2020 Mar 30;9:49. doi: 10.1038/s41377-020-0285-0 (PMC7105458; doi:10.1038/s41377-020-0285-0)
Supplement: Supplementary file 1 — Supplementary Information [file 41377_2020_285_MOESM1_ESM.docx]

**Supplementary Information**

**Enhanced magnetic modulation of light polarization exploiting hybridization with multipolar dark plasmons in magnetoplasmonic nanocavities**

*Alberto López-Ortega^1,^*, Mario Zapata-Herrera^1^, Nicolò Maccaferri^2^, Matteo Pancaldi^3^, Mikel Garcia^1^, Andrey Chuvilin^1,4^, and Paolo Vavassori^1,4,^**

^1^ CIC nanoGUNE, Donostia–San Sebastian 20018, Spain

^2^ Department of Physics and Materials Science, University of Luxembourg, L-1511 Luxembourg, Luxembourg

^3^ Department of Physics, Stockholm University, 106 91 Stockholm, Sweden

^4^ IKERBASQUE, Basque Foundation for Science, Bilbao 48013, Spain

*Correspondence to: p.vavassori@nanogune.eu; [lopezortega.alberto@gmail.com](mailto:lopezortega.alberto@gmail.com)

**
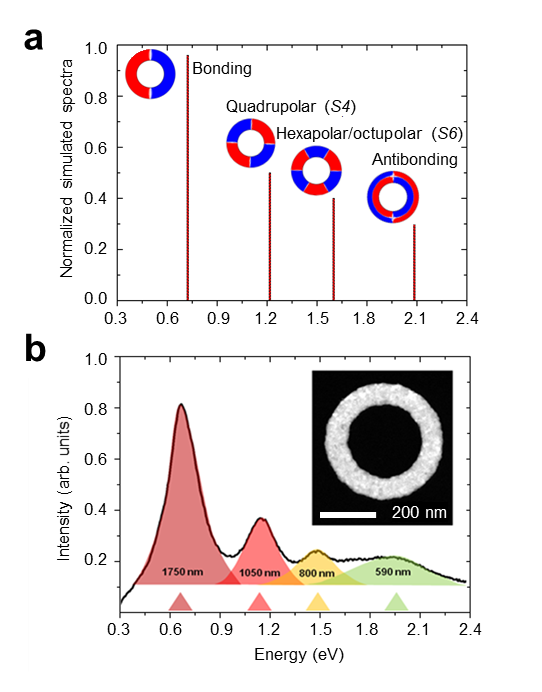
**

**Figure S1** Plasmonic modes of Au nanorings in the Vis-IR spectral region. **a** Theoretical reconstruction of the symmetry of the surface charge density maps and simulated spectrum for an Au ring showing the peaks as returned by the (QNM)-expansion formalism for defect-free structures.^46^ **b** EELS spectrum obtained for an Au ring grown on a SiN_2_ membrane and the respective scanning STEM image.

**
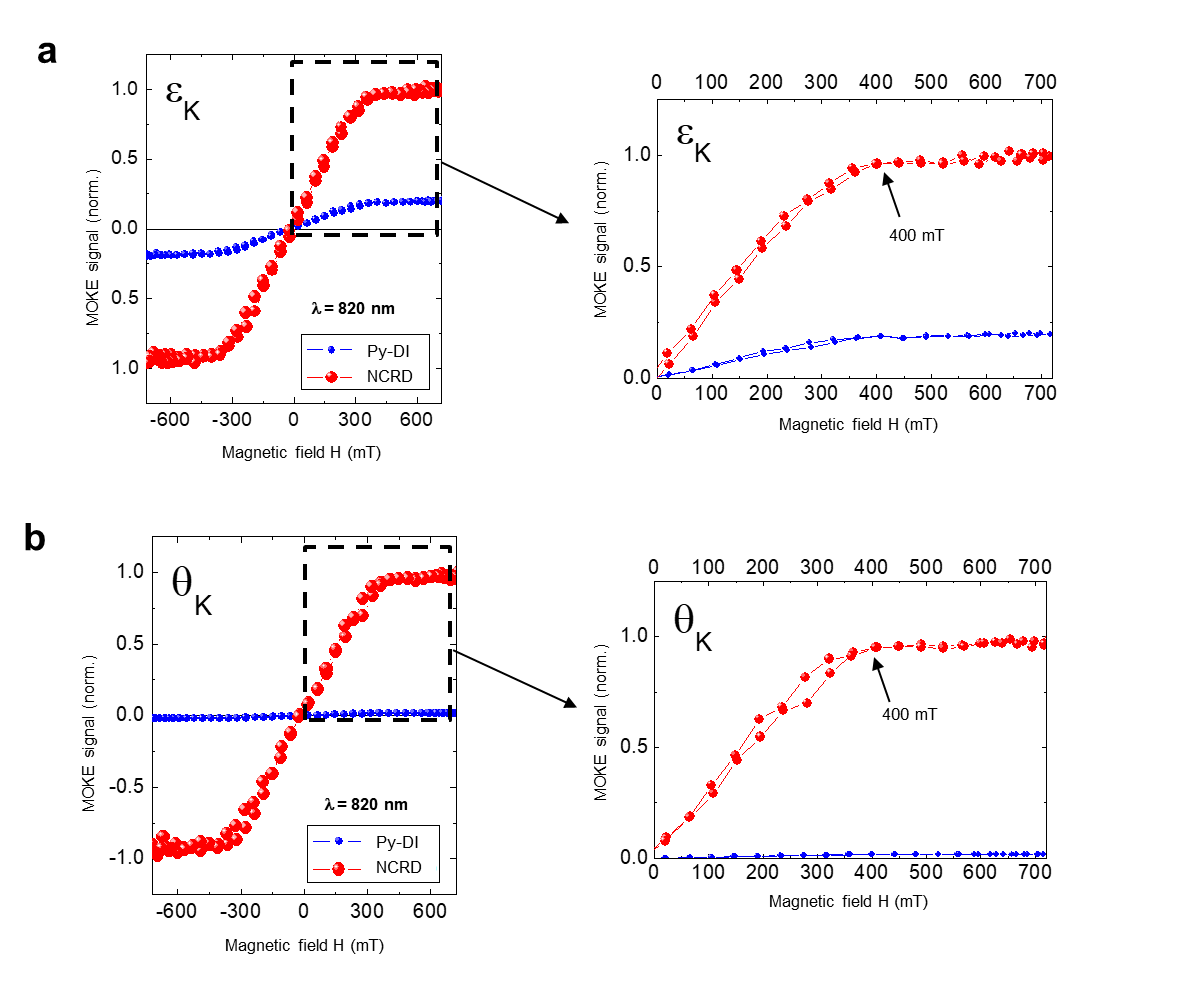
**

**Figure S2** Magnetic field dependent Kerr rotation and ellipticity signals, and their dynamic tunability. a) ε_K_ and b) θ_K_ signals measured at a wavelength of 820 nm as a function of the applied field H for the NCRD and Py-DI. The signals have been normalized to the saturation value (high |H|) for the NCRD. The panels on the right-side show an enlarged portion of the ε_K_ (H) and θ_K_(H) plots (corresponding to the dashed frames in the left-side panels) for a clearer visual evaluation of the saturating field value.

**
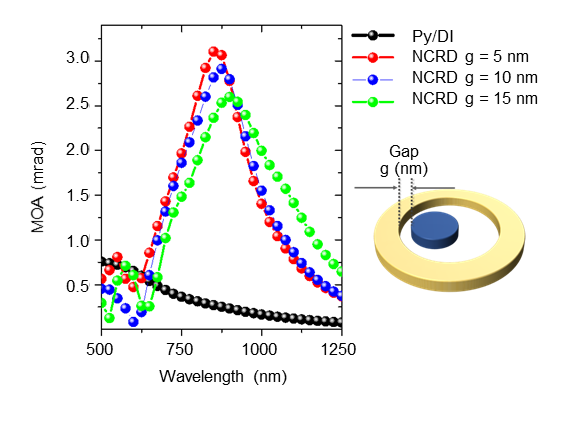
**

**Figure S3** Dependence of the magneto-optical activity on the disk-ring gap size. Simulated MOA spectra as a function of the capacitive coupling, i.e, of the disk-ring gap width (gap), in the NCRD nanocavity. The calculated MOA of the Py-DI nanoantenna is also shown for comparison.


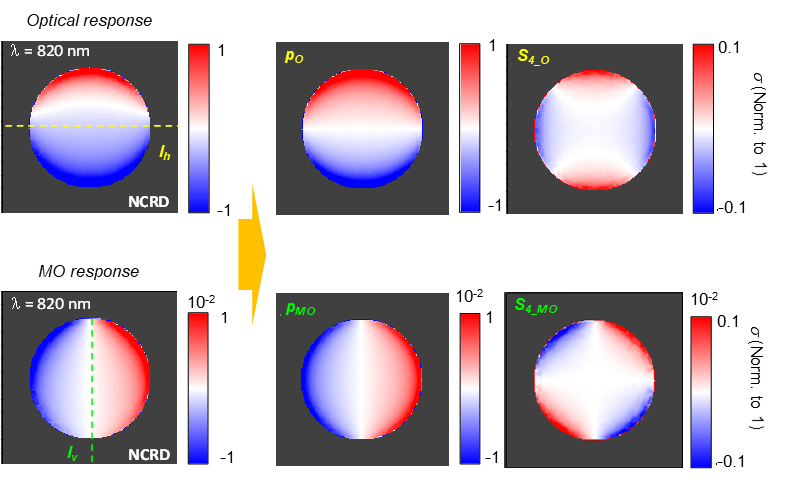


**Figure S4** Multipolar analysis of the optical and magneto-optical response of the Py disk in the NCRD at 820 nm for the NCRD. 2D maps of COMSOL calculated surface charge density *σ* produced by the optical and magnetic-optical activity responses of the Py disk inside the NCRD at the interface between structure and substrate. Intensity is normalized to the maximum value of |*σ*| produced by the optical response (topmost left panel). Imposing a top-bottom and left-right anti-symmetry with respect to the dashed lines l_h_ and l_v_ to the spatial distribution of σ resulting from the optical and magneto-optical responses, respectively, one singles out the optical (*p_O_*) and magnetic-activated (*p_MO_*) electric dipoles. The 10-times-smaller residuals of these symmetry operations display a ***S_4_*** (quadrupolar) symmetry. Interesting, optical and magneto-optical ***S_4_*** modes (***S_4_O_*** and ***S_4_MO_***) are rotated by 45 degrees one respect to the other. The ***S_4_*** modes are π/4 radians out of phase with respect to their respective dipolar mode.


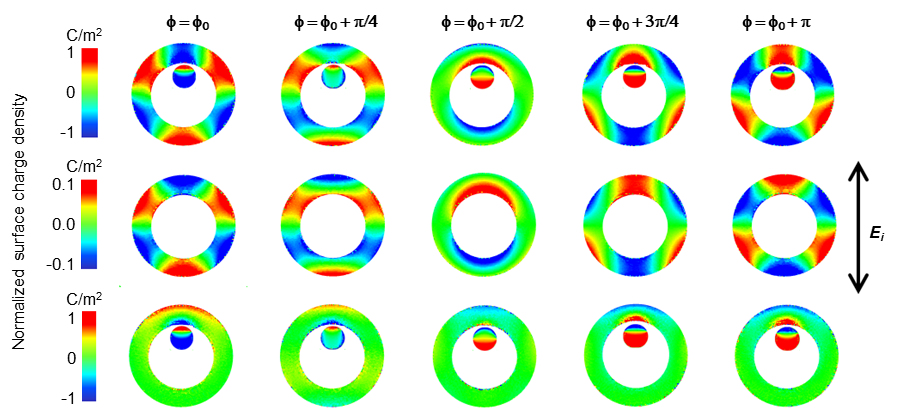


**Figure S5** Surface charge distribution maps at 820 nm for the NCRD and Au-RI in function of time. The topmost sequence shows the NCRD surface charge density maps, normalized to 1, at 820nm and at different values of the phase delay φ (i.e., as a function of time) with respect to an arbitrarily chosen initial phase φ_0_ (i.e., time t_0_) over half-period (φ−φ_0_ [0-π]). The sequence in the middle displays the surface charge density (normalized to the values of the NCRD) map evolution at 820nm for the bare Au-RI. The bottom-most sequence shows the evolution of the difference between the topmost and middle (multiplied by a factor 10) sequences. Simulations were carried out using linearly polarized electromagnetic radiation as indicated by the black arrow (*E_i_* = 1V m^-1^).

**
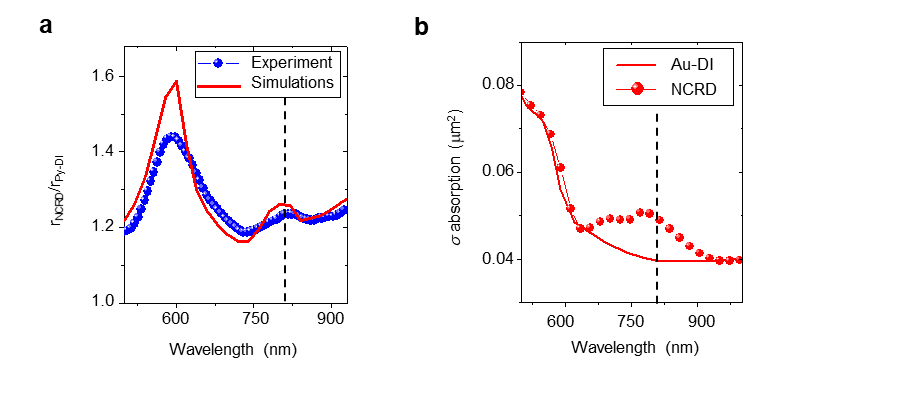
Figure S6** Relative reflectivity of NCRD with respect to Py-DI and absorption cross section with respect to Au-RI. **a** Experimental and simulated spectral dependence of the ratio between the reflectivity of the NCRD (r_NCRD_) and that of the Py-DI (r_Py-DI_). In our experiment, the reflectance signal (R = r^2^) is recorded simultaneously to the MOKE one and utilized to generate the Kerr rotation (θ_K_) and ellipticity (ε_K_) spectra of Fig. 3. **b** Calculated optical absorption cross section spectra for the NCRD and Au_RI (red symbols and line, respectively). The dashed gray lines mark the 820 nm wavelength.


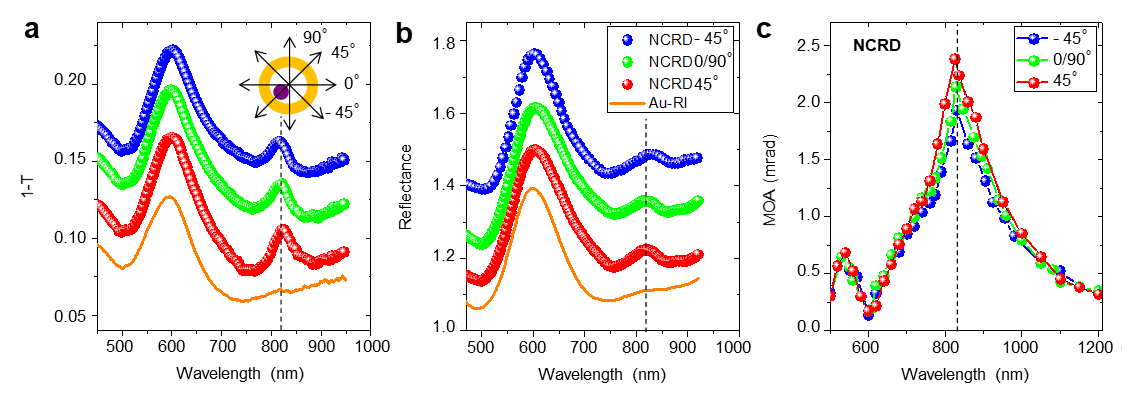


**Figure S7** Optical and MOA spectra at different angles of polarization of the incident light. **a** Extinction (1-T, where T is the transmission) spectra of the NCRD for different angles of the incident polarization. The extinction spectrum of the Au-RI is also shown for comparison. The NCRD spectra are stacked (vertical stacking shift of 0.025) for visualization purposes. **b** Relative reflectance spectra of the NCRD with respect to the glass substrate, for different angles of the incident polarization (reflectance spectrum of the Au-RI is shown for comparison). Also in this case, the NCRD spectra are stacked (vertical stacking shift of 0.1) for visualization purposes. **c** MOA spectra of the NCRD for different angles of the incident polarization. The different angles of polarization are depicted in the inset in Panel **a** and the dashed lines mark the 820 nm wavelength.

**
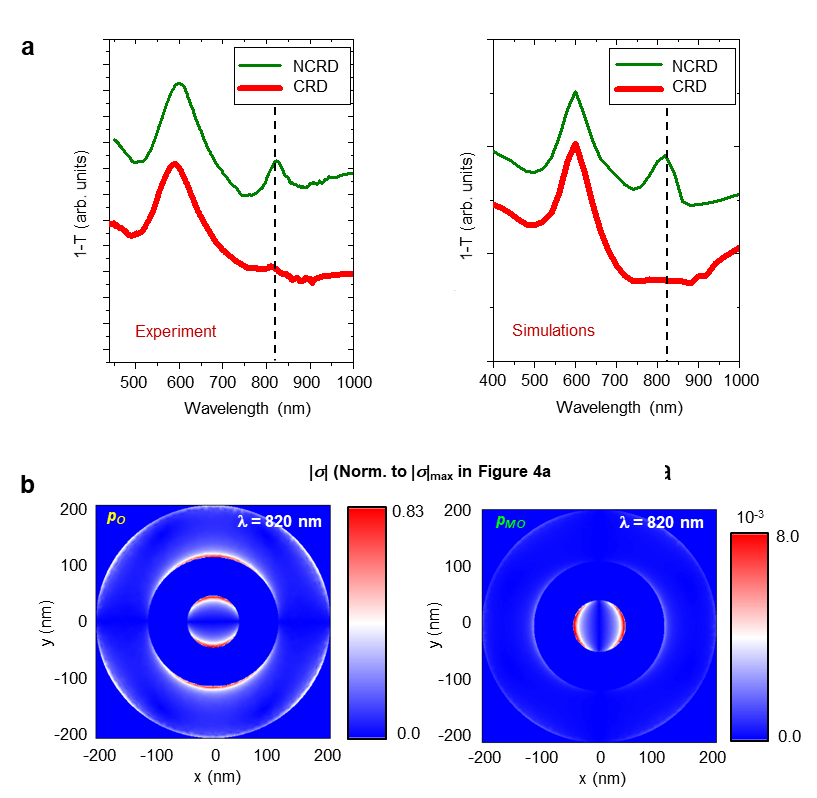
**

**Figure S8** Calculated optical and MO response of CDR cavities at 820 nm. a) Experimental (left) and simulated (right) transmittance spectra for the CRD and NCRD nanocavities. b) 2D maps of COMSOL calculated |*σ*| at 820 nm produced by the optical (*p_O_*) and magneto-activated (*p_MO_*) electric dipoles at the interface between structures and substrate. Intensity is normalized to the maximum value of |*σ*| produced by the optical dipole *p_O_* for the Py-DI at 550nm shown in Figure 4a.
